# Supplementary material for: Quantitative mapping of pseudouridines in bacterial RNA
Source: Nat Commun. 2026 Feb 26;17:3242. doi: 10.1038/s41467-026-70073-3 (PMC13062092; doi:10.1038/s41467-026-70073-3)
Supplement: Supplementary file 1 — Supplementary Information [file 41467_2026_70073_MOESM1_ESM.pdf]

A

Main dataset (read counts)

	WT				$\Psi^{\Delta rRNA}$			
	BS-treated		Untreated		BS-treated		Untreated	
	Replicate 1	Replicate 2	Replicate 1	Replicate 2	Replicate 1	Replicate 2	Replicate 1	Replicate 2
37°C	47,830,011	30,979,915	66,339,301	44,681,214	32,210,846	16,285,713	19,648,523	42,796,684
28°C	41,371,086	Failed	27,361,596	36,338,230	40,198,337	46,136,111	32,009,063	22,044,567
Amp	29,517,607	40,545,856	14,541,768	36,455,055	40,908,949	38,582,249	60,279,104	39,698,535
Gent	44,961,567	48,993,701	25,251,542	37,531,202	42,491,486	51,819,339	24,676,347	25,518,609
NaCl	36,043,144	39,689,999	21,274,520	15,477,345	36,823,100	17,848,545	27,369,861	20,915,579

Additional dataset (read counts)

	BS-treated		Untreated	
	Replicate 1	Replicate 2	Replicate 1	Replicate 2
Δ*truA*	39,401,905	72,704,113	45,734,485	57,170,866
Δ*truB*	65,469,931	63,129,116	93,759,990	47,641,970
Δ*truC*	49,480,995	71,864,809	39,075,824	53,471,468
Δ*truD*	66,282,069	82,034,109	52,464,356	75,127,333

B

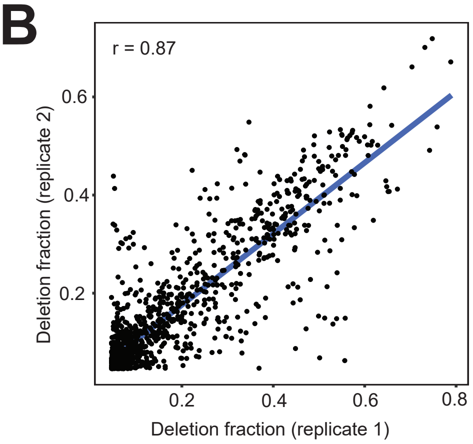

Supplementary Figure 1: Sequencing data and quantitative reproducibility of BS-based Ψ profiling

(A) Total read counts of WT and mutant strains. (B) Pearson correlation between deletion ratios from two biological replicates (*n* = 1,237). Source data are provided as a Source Data file.

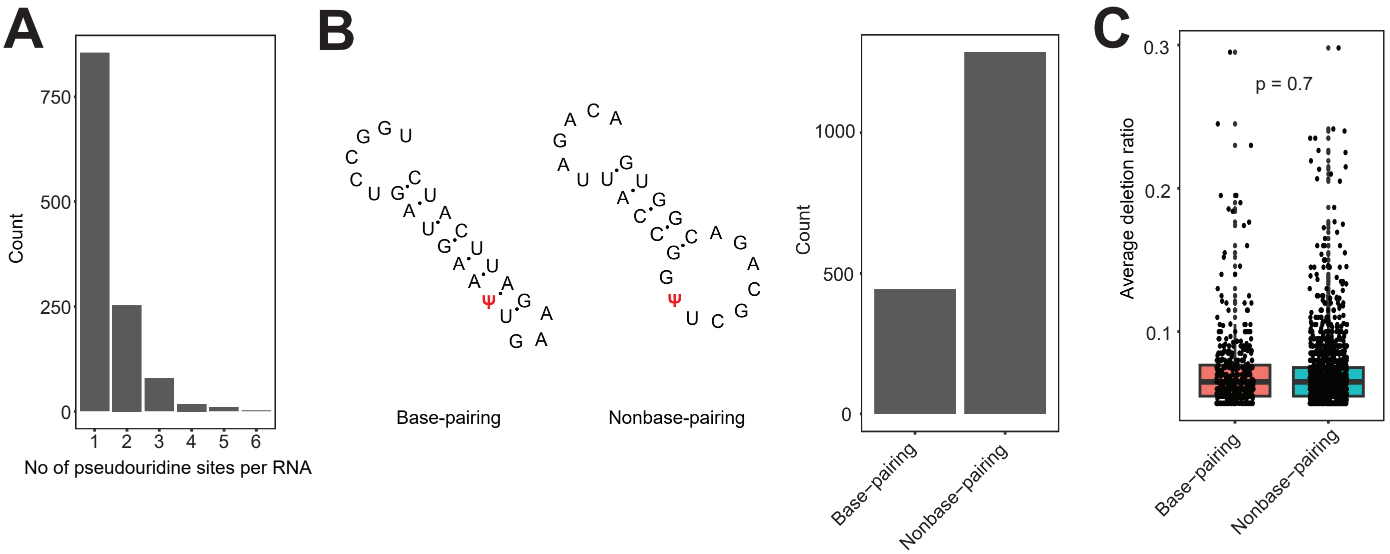

Supplementary Figure 2: Role of mRNA secondary structure on pseudouridylation

(A) Distribution of the number of Ψ sites found in a single mRNA. (B) Examples of base-pairing and nonbase-pairing Ψ sites. The Barplot shows the distribution of pseudouridylation in paired or unpaired uridine residues. (C) Boxplot showing average deletion ratios in paired (*n* = 443) and unpaired Ψ sites (*n* = 1,286). Significant differences between groups were computed with two-sided Wilcoxon rank sum test. Center lines in boxplots represent the median and the edges represent the lower and upper quartiles. Whiskers show values that fall within 1.5x of the interquartile range. Source data are provided as a Source Data file.

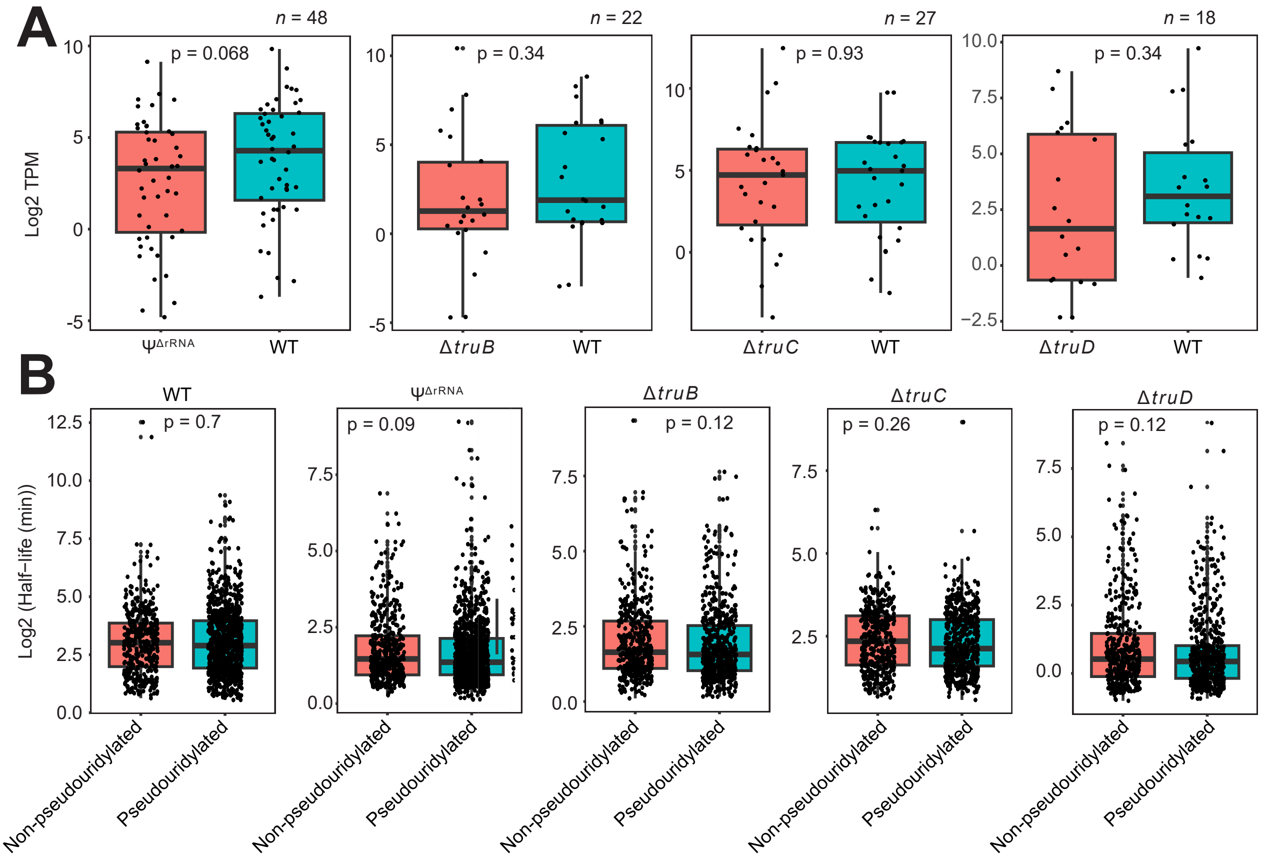

Supplementary Figure 3: Association between RNA pseudouridylation and stability.

(A) Boxplot showing mRNA abundance (TPM) in WT and PUS mutants using random controls from mRNAs with no detectable pseudouridine. Significant differences between groups were computed with two-sided Wilcoxon rank sum test. Center lines in boxplots represent the median and the edges represent the lower and upper quartiles. Whiskers show values that fall within 1.5x of the interquartile range. (B) Boxplot showing global transcript half-lives in WT and PUS mutants from non-pseudouridylated and pseudouridylated RNAs. Significant differences between groups were computed with two-sided Wilcoxon rank sum test. Center lines in boxplots represent the median and the edges represent the lower and upper quartiles. Whiskers show values that fall within 1.5x of the interquartile range. Sample sizes for non-pseudouridylated RNAs: WT, *n* = 424; $\Psi^{\Delta rRNA}$, *n* = 470; Δ*truB, n* = 451; Δ*truC, n* = 456; and Δ*truD, n* = 434. Pseudouridylated RNAs: WT, *n* = 938; $\Psi^{\Delta rRNA}$, *n* = 1,137; Δ*truB, n* = 590; Δ*truC, n* = 588; Δ*truD, n* = 554. Source data are provided as a Source Data file.

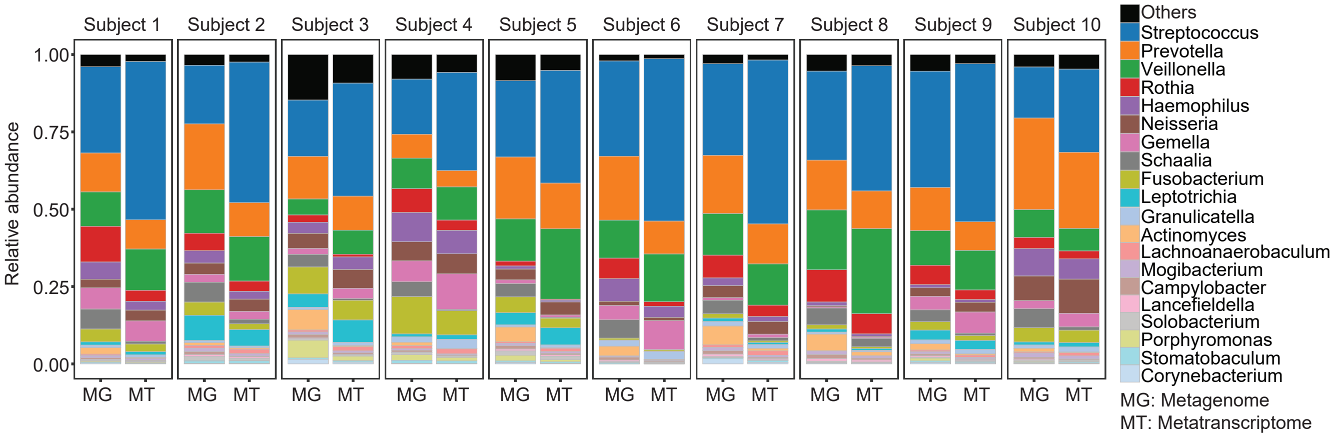

Supplementary Figure 4: Oral microbiome analysis. Comparison of microbial profiles in paired metagenomic and metatranscriptomics oral samples (Belstrøm *et al.*) using Kraken 2/Bracken.
