# Supplementary material for: Quantitative mapping of pseudouridines in bacterial RNA
Source: Nat Commun. 2026 Feb 26;17:3242. doi: 10.1038/s41467-026-70073-3 (PMC13062092; doi:10.1038/s41467-026-70073-3)
Supplement: Supplementary file 2 — Description of Additional Supplementary Files [file 41467_2026_70073_MOESM2_ESM.pdf]

Description of Additional Supplementary Files:

Supplementary Data 1: Pseudouridine profiles in WT and $\Psi^{\Delta rRNA}$ *E. coli* strains under different growth conditions.

Supplementary Data 2: Pseudouridine profiles in *tru E. coli* mutants.

Supplementary Data 3: Sequence motifs for pseudouridylation.

Supplementary Data 4: Pseudouridine profiles in protein-coding RNA from oral microbiome samples. P values were derived from two-sided Fisher's exact test.

Supplementary Data 5: Pseudouridine profiles in 16S rRNA from oral microbiome samples. P values were derived from two-sided Fisher's exact test.

Supplementary Data 6: Bacteria strains used in this study.
