# Supplementary material for: Quantitative mapping of pseudouridines in bacterial RNA
Source: Nat Commun. 2026 Feb 26;17:3242. doi: 10.1038/s41467-026-70073-3 (PMC13062092; doi:10.1038/s41467-026-70073-3)
Supplement: Supplementary file 4 — Reporting Summary [file 41467_2026_70073_MOESM4_ESM.pdf]

nature portfolio

Corresponding author(s): Akintunde Emiola

Last updated by author(s): Jan 29, 2026

Reporting Summary

Nature Portfolio wishes to improve the reproducibility of the work that we publish. This form provides structure for consistency and transparency in reporting. For further information on Nature Portfolio policies, see our Editorial Policies and the Editorial Policy Checklist.

Statistics

For all statistical analyses, confirm that the following items are present in the figure legend, table legend, main text, or Methods section.

n/a	Confirmed	
☐	☒	The exact sample size (*n*) for each experimental group/condition, given as a discrete number and unit of measurement
☐	☒	A statement on whether measurements were taken from distinct samples or whether the same sample was measured repeatedly
☐	☒	The statistical test(s) used AND whether they are one- or two-sided<br>*Only common tests should be described solely by name; describe more complex techniques in the Methods section.*
☒	☐	A description of all covariates tested
☐	☒	A description of any assumptions or corrections, such as tests of normality and adjustment for multiple comparisons
☐	☒	A full description of the statistical parameters including central tendency (e.g. means) or other basic estimates (e.g. regression coefficient) AND variation (e.g. standard deviation) or associated estimates of uncertainty (e.g. confidence intervals)
☐	☒	For null hypothesis testing, the test statistic (e.g. *F*, *t*, *r*) with confidence intervals, effect sizes, degrees of freedom and *P* value noted<br>*Give P values as exact values whenever suitable.*
☒	☐	For Bayesian analysis, information on the choice of priors and Markov chain Monte Carlo settings
☒	☐	For hierarchical and complex designs, identification of the appropriate level for tests and full reporting of outcomes
☐	☒	Estimates of effect sizes (e.g. Cohen's *d*, Pearson's *r*), indicating how they were calculated

Our web collection on statistics for biologists contains articles on many of the points above.

Software and code

Policy information about availability of computer code

Data collection	No software was used to collect data
Data analysis	Reads were mapped using bwa-mem v0.7.17. Mapped reads were realigned using ABRA2 to improve detection of indels in downstream analysis. Bam-readcount v1.0.1 was used to retrieve nucleotide coverage and sequence variant information and the resulting output was subsequently parsed using brc-parser.py

For manuscripts utilizing custom algorithms or software that are central to the research but not yet described in published literature, software must be made available to editors and reviewers. We strongly encourage code deposition in a community repository (e.g. GitHub). See the Nature Portfolio guidelines for submitting code & software for further information.

Data

Policy information about availability of data

All manuscripts must include a data availability statement. This statement should provide the following information, where applicable:

- Accession codes, unique identifiers, or web links for publicly available datasets
- A description of any restrictions on data availability
- For clinical datasets or third party data, please ensure that the statement adheres to our policy

Raw sequence reads generated in this study were deposited in Sequence Reads Archive (SRA) under BioProject accession number PRJNA1414121 [https://www.ncbi.nlm.nih.gov/bioproject/?term=PRJNA1414121]. The oral isolates and metagenome-assembled genomes (MAGs) were downloaded from the Human Reference Oral Microbiome (HROM) database (https://www.decodebiome.org/HROM/listdir.php?directory=data/genome_catalog). For paired oral metagenomics

and metatranscriptomics analysis (BioProject PRJNA396840 [https://www.ncbi.nlm.nih.gov/bioproject/396840]), the following SRA accessions were used; SRR5892221, SRR5892220, SRR5892219, SRR5892218, SRR5892225, SRR5892224, SRR5892223, SRR5892222, SRR5892227, SRR5892226, SRR5892199, SRR5892198, SRR5892197, SRR5892196, SRR5892203, SRR5892202, SRR5892201, SRR5892206, SRR5892194, SRR5892193. All other necessary data are included in the Supplementary Information.

Research involving human participants, their data, or biological material

Policy information about studies with human participants or human data. See also policy information about sex, gender (identity/presentation), and sexual orientation and race, ethnicity and racism.

Reporting on sex and gender	N/A
Reporting on race, ethnicity, or other socially relevant groupings	N/A
Population characteristics	The study included 18 participants: 13 healthy volunteers and 5 patients with chronic periodontitis. This cohort (56% female, 44% male) had a mean age of 36.7 years (SD = 9.9; range 23–57). All participants were confirmed negative for Hep B, Hep C, HIV, diabetes, and had no history of autoimmune disorders. Self-reported race and ethnicity data were collected to describe cohort diversity and were not used as a proxy for biological variation.
Recruitment	Participants were recruited from within the general Bethesda, Maryland surrounding metropolitan area and screened for inclusion/exclusion criteria. All study participants provided written informed consent for participation in this study. Beyond healthy volunteer selection, based on protocol inclusion/exclusion criteria, we also included a subset of individuals with severe periodontitis (stage III/IV) so as to include a subset of individuals with a periodontitis-associated microbiome.
Ethics oversight	Collection of human samples was performed on an IRB clinical protocol approved by the National Institutes of Health (NIH) Clinical Center (ClinicalTrials.gov ID NCT01568697). All study participants provided written informed consent for participation in this study.

Note that full information on the approval of the study protocol must also be provided in the manuscript.

Field-specific reporting

Please select the one below that is the best fit for your research. If you are not sure, read the appropriate sections before making your selection.

[x] Life sciences [ ] Behavioural & social sciences [ ] Ecological, evolutionary & environmental sciences

For a reference copy of the document with all sections, see nature.com/documents/nr-reporting-summary-flat.pdf

Life sciences study design

All studies must disclose on these points even when the disclosure is negative.

Sample size	To identify pseudouridine landscape in bacteria RNAs, we sequenced 120 samples. Previous studies have identified pseudouridines in humans, yeast, and bacteria by analyzing significantly fewer dataset.
Data exclusions	Only data not meeting pre-determined threshold cutoff were excluded. For instance, our analysis required a minimum of 20X coverage depth.
Replication	Replicates were conducted for the E. coli pseudouridine analyses and successfully reproduced.
Randomization	Random control sets of equal size were analyzed to determine the link between pseudouridine and mRNA abundance
Blinding	Blinding was not possible as the individual performing the experiments typically prepared the samples being measured

Reporting for specific materials, systems and methods

We require information from authors about some types of materials, experimental systems and methods used in many studies. Here, indicate whether each material, system or method listed is relevant to your study. If you are not sure if a list item applies to your research, read the appropriate section before selecting a response.

Materials & experimental systems

n/a	Involved in the study
☒	☐ Antibodies
☒	☐ Eukaryotic cell lines
☒	☐ Palaeontology and archaeology
☒	☐ Animals and other organisms
☐	☒ Clinical data
☒	☐ Dual use research of concern
☒	☐ Plants

Methods

n/a	Involved in the study
☒	☐ ChIP-seq
☒	☐ Flow cytometry
☒	☐ MRI-based neuroimaging

Clinical data

Policy information about clinical studies
All manuscripts should comply with the ICMJE guidelines for publication of clinical research and a completed CONSORT checklist must be included with all submissions.

Clinical trial registration	ClinicalTrials.gov ID NCT01568697
Study protocol	Oral Bacteria and Immune System Problems Involved in Gum Disease
Data collection	Data and sample collection performed at National Institutes of Health Clinical Center (CC) (National Institute of Dental and Craniofacial Research (NIDCR))
Outcomes	cross sectional/natural history protocol designed to investigate the clinical, microbiologic, and immunologic consequences of genetic immune defects in the oral cavity.

Plants

Seed stocks	N/A
Novel plant genotypes	N/A
Authentication	N/A
